# Psychotropic Medication Use in Children and Youth with Autism Enrolled in Medicaid

**DOI:** 10.1007/s10803-023-06182-5

**Published:** 2023-12-18

**Authors:** Jessica E. Rast, Sha Tao, Whitney Schott, Lindsay L. Shea, Edward S. Brodkin, Connor M. Kerns, Charles E. Leonard, Michael J. Murray, Brian K. Lee

**Affiliations:** 1 A.J. Drexel Autism Institute, Drexel University, Philadelphia, PA USA; 2 Department of Psychiatry, Perelman School of Medicine, University of Pennsylvania, Philadelphia, PA USA; 3 Department of Psychology, University of British Columbia, Vancouver, BC Canada; 4 Center for Real-World Effectiveness and Safety of Therapeutics, Perelman School of Medicine, University of Pennsylvania, Philadelphia, PA USA; 5 Center for Clinical Epidemiology and Biostatistics, Perelman School of Medicine, University of Pennsylvania, Philadelphia, PA USA; 6 Department of Biostatistics, Epidemiology, and Informatics, Perelman School of Medicine, University of Pennsylvania, Philadelphia, PA USA; 7 Leonard Davis Institute of Health Economics, University of Pennsylvania, Philadelphia, PA USA; 8 Department of Psychiatry, College of Medicine, Pennsylvania State University, Hershey, PA USA; 9 Department of Epidemiology and Biostatistics, Drexel University Dornsife School of Public Health, Philadelphia, USA

**Keywords:** Psychotropic medication, Medicaid, Children and youth with autism, Polypharmacy

## Abstract

Children with autism frequently present with complex mental health diagnoses and psychotropic medications are often a component of comprehensive biopsychosocial treatment plans for these conditions. The purpose of this study is to provide rates and patterns of psychotropic medication use, and predictors thereof, in children and youth with autism enrolled in Medicaid across the US. This study examined national Medicaid claims from 2008 to 2016 of all children and youth with autism ages 0–21 years enrolled in Medicaid. Psychotropic medication use was examined across several child and youth characteristics, including age, co-occurring mental health conditions, sex, and race and ethnicity. About half of children and youth with autism enrolled in Medicaid had at least one psychotropic prescription in a year, a number that decreased slightly across the study period due to decreases in the prescription of antipsychotics. As new medications for autism or co-occurring conditions are developed and deployed, and as the understanding of the characteristics of the population of children with autism evolves, studying rates of medication usage helps to understand utilization patterns and differences in access to quality care.

**Supplementary Information:**

The online version contains supplementary material available at 10.1007/s10803-023-06182-5.

## Introduction

Children with autism frequently present with complex mental health diagnoses. As many as 78% of children with autism ages 3–17 years have ≥ 1 co-occurring mental health condition, with 49% having two or more (Kerns et al., 2020). The most common challenges include aggression and/or self-injury (61%); attention-deficit/hyperactivity disorder (ADHD) (48%); anxiety (40%); and depression (16%) (Kerns et al., 2020; Rast et al., 2021). Psychotropic medications are often a component of comprehensive biopsychosocial treatment plans for these conditions, thus research on their use is warranted (American Academy of Child Adolescent Psychiatry (AACAP), 2015). Psychotropic medications describe a broad range of drugs used in the treatment of psychiatric and neurological disorders that affect brain function, behavior, mood, thoughts, and/or perception. Several classes of psychotropic medications are used to treat mental health concerns, including antidepressants, antipsychotics, antianxiety medications, sedatives, hypnotics, stimulants, and antiseizure medications used as mood stabilizers.

The high rates of co-occurring mental health conditions and interfering behavioral symptoms in children with autism may drive the use of polypharmacy (Jobski et al., 2017), with 35% of children taking medications from ≥ 2 psychotropic classes concurrently, and 15% taking medications from ≥ 3 classes (Spencer et al., 2013b). In children with autism, the presentation of complex symptom profiles makes prescription and symptom management a difficult task that is often approached on a trial-and-error basis. As the population of autistic children and awareness of the need for safe and effective pharmacological behavioral health interventions have grown, more studies have examined the safety and efficacy of psychotropic medications in autistic populations (de Pablo et al., 2023; Henneberry et al., 2021; LeClerc & Easley, 2015; Persico et al., 2021). Psychotropic medication should ideally be prescribed as part of a management plan including evidence-based psychosocial interventions, a commitment to youth- and family-centered care, and the use of trauma-informed care principles (AACAP, 2015; Walkup, 2009). Ongoing monitoring of symptoms, safety, and side effects is imperative. Only two psychotropic medications are approved by the U.S. Food and Drug Administration for children with autism (risperidone and aripiprazole, for irritability or aggression). Understanding the profile of use of such medications off-label for autism symptoms versus co-occurring conditions can help to understand the clinical decisions that are being made in behavioral health care.

A few studies of national estimates of psychotropic medication use in children with autism are based on data from the early 2000s (Mandell et al., 2008; Schubart et al., 2014; Spencer et al., 2013a). They find high use overall (64% of autistic youth ages 3–17 years enrolled in Medicaid 2000–2003) (Schubart et al., 2014), with the highest usage in older autistic youth (73% of youth ages 18–21 years enrolled in Medicaid in 2001) and lower usage in younger children (18% of children ages 0–2 years) (Mandell et al., 2008). Other studies have examined convenience samples and found similarly high rates of use in older youth with autism (64% of autistic youth ages 12–17 years had at least one psychotropic medication compared to 10% of children 3–5 years) (Coury et al., 2012).

As new medications for autism or co-occurring conditions are developed and deployed, and as the understanding of the characteristics of the population of children with autism evolves, studying rates of medication usage helps to understand utilization patterns and differences in access to quality care. These medications often have significant neurological, behavioral, or somatic side effects and there is a general lack of data to understand their use as treatments among autistic children. This study provides estimates of psychotropic medication use, and predictors thereof, in children with autism enrolled in Medicaid across the US from 2008 to 2016.

## Methods

### Data and Study Population

Medicaid claims were used to conduct retrospective descriptive analyses of autistic children enrolled in Medicaid between 2008 and 2016 (n = 942,125). Data were extracted from Medicaid Analytic eXtract (MAX) files and Transformed Medicaid Statistical Information System (T-MSIS) Analytic Files (TAF) between 2008 and 2016. Children with autism ages 0–21 enrolled in Medicaid in all 50 US states and Washington, DC were included. Youth were required to be enrolled for no less than 9 months in a year to be included in that year’s analysis and have at least one inpatient claim or two other claims associated with an autism diagnostic code (ICD-9 299.xx, ICD-10 F84.x), in alignment with the Chronic Condition Warehouse algorithm for detecting autism in claims data. The positive predictive value of an autism diagnosis within healthcare claims in identifying autistic children is very high (97%-98% of children classified with autism in this way actually have autism) (Burke et al., 2014; Dodds et al., 2009; Fombonne et al., 2004). In acknowledgement of language choices that may be preferred by autistic people, we have used the term “autism” in this study, as opposed to the more clinical, but potentially divisive, “autism spectrum disorder”(Monk et al., 2022).

### Outcomes: Psychotropic Medication Prescriptions Filled

We examined several patterns of concurrent medication use of individual prescriptions: (1) use of two or more prescriptions for 60 + days, (2) use of three or more prescriptions for 60 + days, (3) use of two or more prescriptions for 90 + days, and (4) use of three or more prescriptions for 90 + days. Two prescriptions is a common definition of polypharmacy in children, and 60 and 90 days were chosen to ensure children were using more than one medication concurrently and not experiencing medication changes, as records capture pharmacy dispensings but not necessarily medication use (Horace et al., 2020; Lohr et al., 2018). We required that two or three prescriptions were filled for different medications that, if taken as directed, would be used at the same time for at least 60 or 90 consecutive days. Prescriptions came from five classes of psychotropic medications: antidepressants; antipsychotics; anxiolytics, sedative, and hypnotics; stimulants; and anticonvulsants.

### Characteristics

Characteristics of autistic youth included age, sex, race/ethnicity, criteria for Medicaid enrollment, urbanicity, and presence of mental and behavioral co-occurring diagnoses. Co-occurring mental and behavioral diagnoses included anxiety disorders; depressive disorders; bipolar; ADHD, conduct disorders, and hyperkinetic syndrome; and schizophrenia or other psychotic disorders (eTable1 for ICD-9 and ICD-10 codes). Age was calculated at the end of the year, and the number rounded down to the nearest integer (age 5.9 is rounded to age 5 years).

### Statistical Analysis

We first examined the prevalence of psychotropic medication use by class and overall cross sectionally for each study year. Within each year, we examined the prevalence of the patterns described above for six or more months in the year. We then examined use in each year by age in years (0–5, 6–11, 12–17, and 18–21). The prevalence of the use of two or more medications for 60 or more days, use of three or more medications for 60 or more days, use of two or more medications for 90 or more days, use of three or more medications for 90 days or more, and the average number of months with three or more medications in a given year were calculated. The prevalence of each class of medication was calculated by mental and psychiatric co-occurring diagnoses. Finally, we presented the prevalence of medication use by each characteristic and the adjusted relative risk (RR) using modified Poisson regression, which accounts for clustering using robust standard errors and presents a RR which can be interpreted directly as a risk (as opposed to an odds ratio). We examined factors associated with (1) any psychotropic medication use, and (2) use of 3 or more psychotropic medications for at least 90 consecutive days in autistic youth enrolled in Medicaid in 2016. We chose these outcomes as they represent the lowest and highest level of psychotropic medication use identified in this study. Each model adjusted for the characteristics described above and state. We repeated each model for 2008, 2012, and 2016 to assess potential differences over the study period but present the 2016 results as no meaningful differences were identified. Analysis was conducted in SAS version 9.4.

## Results

**Fig. 1:**
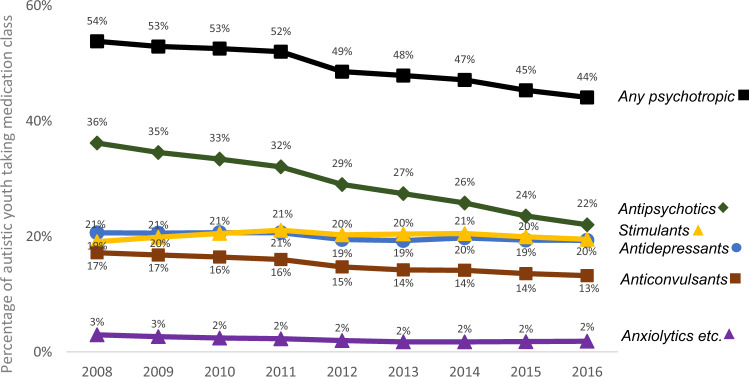
Repeated cross-sectional prevalence of psychotropic prescriptions over time in all autistic youth

In any given year, about half of autistic youth filled a psychotropic prescription (Fig. 1), a percentage that decreased over the study period (2008–2016) from 54 to 44%. In all years, the most common class of psychotropic was antipsychotics, but the percentage of autistic youth prescribed an antipsychotic decreased from 36% in 2008 to 22% in 2016. The least common class of psychotropic medication was anxiolytics, sedatives, and hypnotics, which 3% or less of autistic youth were prescribed in a given year.

### Patterns of Medication Use

**Fig. 2:**
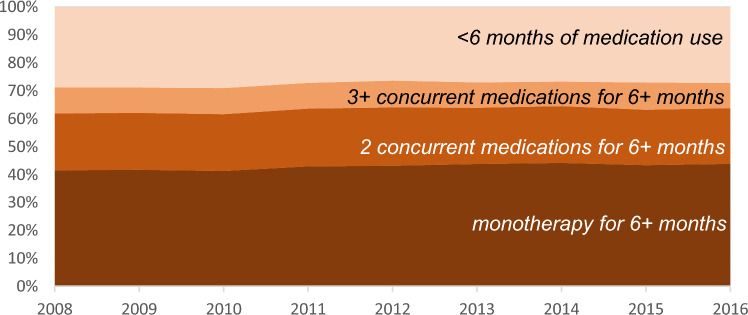
Psychotropic medication patterns in autistic youth with at least one prescription

Figure 2 examines patterns of psychotropic medication use for autistic youth who had at least one prescription across study years. Medication patterns were consistent over study years. Monotherapy for at least 6 months in the year was seen in 40% of autistic youth across the study period (41.5% in 2008 and 43.8% in 2016). Slightly over one-quarter of autistic youth who were prescribed at least one psychotropic medication in a given year took medication for less than six months (28.8% in 2008 and 27.1% in 2016). Concurrent use of two medications for at least six months was seen in 20.4% of autistic youth in 2008 and in 20.0% in 2016. Concurrent use of three or more medications was present in 9.3% of autistic youth in 2008 and 9.1% of autistic youth in 2016.

Table 1 examines concurrent medication use in autistic youth in 2016 by age group. Concurrent medication use was not common in autistic children ages 0–5 years. Concurrent medication use was most common in autistic youth ages 18–21 years; over one-third of autistic youth of ages 18–21 took two or more medications concurrently for at least 60 days (36.8%) and nearly as many took two or more medications for at least 90 days (34.2%). Concurrent use of at least three medications for at least 60 days (16.6%) or 90 days in a year (14.8%) was less common than use of two or more medications in the oldest autistic youth. Of youth participating in concurrent use of three or more medications, the average number of months in a year was highest in the oldest youth, an average of 4.7 months of 12.

**Table 1:**
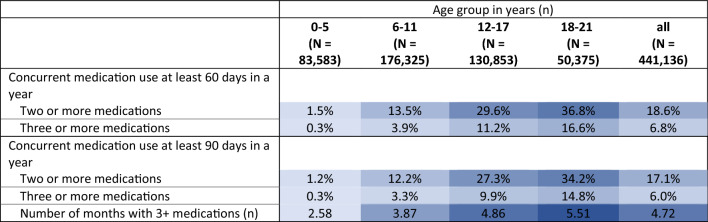
Multiple psychotropic prescription in autistic youth 2016

Autistic youth with certain co-occurring mental or behavioral diagnoses had higher rates of psychotropic prescription than autistic youth without these conditions (Table 2). Youth with autism and schizophrenia or another psychotic disorder were the most likely to have psychotropic prescriptions, of the class antipsychotics (88.4%). Autistic youth with bipolar also commonly had antipsychotic prescriptions over the study period (83.9%), while these were not common in autistic youth with none of these diagnoses (11.1%). Stimulants were prescribed in about half of autistic youth with any of the diagnoses seen in Table 3, but infrequently prescribed in youth without a co-occurring diagnoses (5.5%). Estimates by year show a similar pattern over the study period (eTable 2).

**Table 2:**
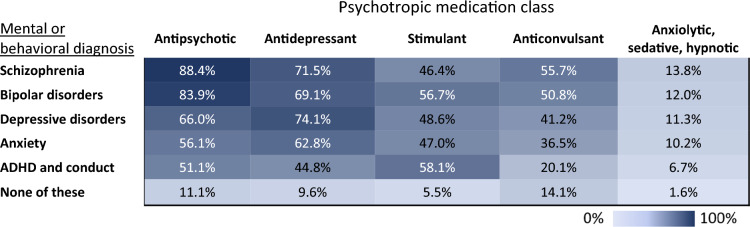
Psychotropic classes prescribed to autistic youth by co-occurring diagnoses (combined 2008-2016)

**Table 3:** Risk of psychotropic medication use & concurrent 3 + medication use in autistic youth in 2016

	All youth		Any psychotropic medication			Concurrent 3 + medication use		
	N	Col %	Prevalence (%)	aRR	95% CI	Prevalence (%)	aRR	95% CI
Age in 2016								
0–5 years	83,583	18.95	10.27	0.25	(0.24,0.25)	0.25	0.05	(0.04,0.05)
6–11 years	176,325	39.97	41.51	0.75	(0.74,0.75)	3.27	0.41	(0.4,0.42)
12–17 years	130,853	29.66	60.88	ref		9.87	ref	
18–21 years	50,375	11.42	65.85	1.15	(1.14,1.16)	14.82	1.52	(1.48,1.56)
Sex								
Female	91,909	20.83	43.76	1.03	(1.02,1.03)	6.73	1.16	(1.12,1.19)
Male	349,227	79.17	44.21	Ref	5.78	ref		
Race								
American Indian and Alaska Native	3,503	0.79	41.79	0.84	(0.81,0.87)	5.80	0.76	(0.67,0.87)
Asian/Hawaiian/Pacific Islander	14,089	3.19	24.80	0.68	(0.66,0.69)	2.04	0.42	(0.38,0.47)
Black	59,142	13.41	39.41	0.88	(0.87,0.89)	4.07	0.64	(0.62,0.67)
Hispanic/Latino	89,232	20.23	32.21	0.79	(0.79,0.8)	2.86	0.55	(0.53,0.58)
More than one	5,711	1.29	53.07	1.04	(1.01,1.06)	7.65	1.01	(0.93,1.1)
White	209,787	47.56	52.34	Ref		8.16	Ref	
Missing	59,672	13.53	41.51	0.91	(0.9,0.92)	5.61	0.80	(0.77,0.83)
Eligibility								
Poverty	122,119	27.68	40.15	0.92	(0.91,0.93)	4.25	0.74	(0.72,0.76)
Disability	230,205	52.18	47.50	ref		7.00	ref	
Other	87,661	19.87	40.54	0.95	(0.94,0.95)	5.59	0.90	(0.87,0.93)
Missing	1,151	0.26	60.38	1.01	(0.97,1.05)	13.81	1.30	(1.13,1.5)
Urbanicity								
Urban	325,340	73.75	41.18	Ref	5.28	Ref		
Suburban	93,145	21.11	52.92	1.09	(1.09,1.1)	8.00	1.17	(1.14,1.21)
Rural	16,085	3.65	53.09	1.07	(1.06,1.09)	8.72	1.23	(1.17,1.3)
Missing	6,566	1.49	42.81	0.99	(0.96,1.01)	4.86	0.82	(0.74,0.91)
Co-occurring diagnoses								
Anxiety	54,491	12.35	76.73	1.26	(1.26,1.27)	14.00	1.41	(1.37,1.45)
Depression	19,605	4.44	84.04	1.07	(1.06,1.08)	17.39	1.06	(1.02,1.1)
Bipolar disorder	26,937	6.11	91.57	1.23	(1.22,1.24)	23.45	2.04	(1.98,2.1)
ADHD, conduct disorders	139,046	31.52	76.16	2.09	(2.08,2.1)	11.20	2.19	(2.13,2.24)
Schizophrenia	9,175	2.08	92.05	1.11	(1.10,1.12)	22.98	1.28	(1.23,1.34)
None of these	265,198	60.12	23.92	–		2.57	–	
Number of months enrolled, mean (SD)	11.93	0.38	11.95 (0.31)	NA		11.98 (0.22)	NA	

*aRR* adjusted relative risk, as estimated by Poisson regression with all covariates in the table included simultaneously excluding number of months enrolled

### Medication Use in Young Children

In 2016, 10% of children with autism ages 0–5 years had a prescription for at least one psychotropic medication (Table 3). Most of these children were ages 3–5 years, but 12% were ages 0–2 years. In these children, the most common prescriptions were levetiracetam, diazepam, and oxcarbazepine, which have on-label uses as treatments for seizures or epilepsy and most of these children had an epilepsy diagnosis (data in eTable 3).

### Rates of Medication Use and Adjusted Relative Risk

Psychotropic prescription was more common in some autistic youth in 2016, including older youth and those with co-occurring mental or behavioral diagnoses (Table 3). The prevalence of any psychotropic prescription in children ages 0–5 years was 10%, compared to 66% in youth ages 18–21 years, and anticonvulsants were the most common psychotropic in the youngest children. About one-quarter (24%) of autistic youth with no co-occurring mental or behavioral conditions took any psychotropic, compared to 92% of youth with schizophrenia or bipolar disorder.

Table 3 also presents relative risk of (1) any psychotropic medication and (2) use of three or more psychotropic medications for 90 days or more by demographic and clinical characteristics seen in the table in 2016. Autistic youth with co-occurring mental and behavioral conditions had the highest risk of psychotropic medication compared to youth without the condition, with ADHD (aRR 2.09, 95%CI 2.08,2.10) and anxiety disorders (aRR 1.26, 95%CI 1.26,1.27) having the largest magnitude risk with use of any psychiatric medication. Similar to use of any psychotropic medication, youth with certain co-occurring mental health and behavioral conditions had highest risk of use of three or more prescriptions for at least 90 consecutive days, including ADHD (aRR 2.19, 95%CI 2.13,2.24) and bipolar disorders (aRR 2.04, 95%CI 1.98, 2.10). In comparison to white autistic children, Black, Asian/Hawaiian/Pacific Islander, Hispanic, and American Indian and Alaska Native children had lower risk of any psychotropic prescription, and even further reduced risk of concurrent medication use (3 + prescriptions). Estimates followed similar trends over the study period (eTables 4 and 5).

## Discussion

About half of children and youth with autism enrolled in Medicaid from 2008 to 2016 had at least one psychotropic prescription in a year, a number that decreased across the study period. Previous studies from earlier years, smaller samples, and shorter ranges reported one-quarter (Coury et al., 2012) to two-thirds (Schubart et al., 2014; Spencer et al., 2013a) of autistic youth with psychotropic use. Our overall finding of any psychotropic use was similar to a study examining Medicaid enrolled children in 2001 (Mandell et al., 2008) and another examining children enrolled in several Kaiser Permanente networks in 2010 (both privately and publicly insured children) (Madden et al., 2017). Eligibility for Medicaid may be determined by income (households making below a certain income threshold) or disability status. Most children with autism enrolled in Medicaid in these study years were found eligible under the disability category. While children enrolled in Medicaid are not representative of all children, about half of all children with autism are insured by public health insurance, including Medicaid (Rast et al., 2020). Further, studies in other populations have found that medication use may be similar for children regardless of insurance type (private insurance versus Medicaid-enrolled children) (Ali et al., 2019).

The decrease seen over this study period was driven by a decrease specifically in prescribing of antipsychotics. Prior research has reported a decrease in antipsychotic use in autistic children over the same time period in the UK (Alfageh et al., 2020). Decreases in prescription may be attributed to monitoring programs and changes in provider behaviors; risperidone and aripiprazole, the two antipsychotics with FDA approval for use in autism for irritability and aggression, are increasingly considered last resorts for crisis behaviors, to be used after or in conjunction with behavioral interventions. Changes to insurance reimbursement policies that require prior authorization for antipsychotic prescriptions in children have been shown to impact a small decrease in their use over this period (Stein et al., 2014).

Certain youth characteristics were related to increased risk of polypharmacy. The presence of co-occurring diagnoses increased the risk of concurrent use of three or more medications, especially ADHD and bipolar disorders. Complexity of condition management with co-occurring diagnoses or an interaction of these conditions with autism may drive polypharmacy. Race and ethnicity were also related to risk of concurrent medication use, where American Indian and Alaska Native, Asian/Hawaiian/ Pacific Islander, Black, and Hispanic children had decreased use of polypharmacy compared to white children. Prior studies have shown increased use of psychotropic medication in white children compared to children of another race or ethnicity (Lê Cook et al., 2017; Logan et al., 2015). It is unknown if minoritized children are not offered prescriptions or if their caregivers are less likely to accept such a treatment approach.

Findings also point toward the need for assessing opportunities for access to behavioral services. The prescription of psychotropic medications to one-quarter of children with autism without co-occurring conditions suggests a need for bolstered treatment planning. As most medications are not approved for treatment of autism related symptoms, such use could lead to safety concerns or complex needs among families and caregivers. Alternatively, it could indicate undiagnosed conditions that may be challenging to identify and treat. High rates of concurrent medication use may underscore the lack of available treatments and accessible service delivery programs among autistic youth, especially in communities at higher risk for health inequities.

The use of psychotropic medications is a priority for understanding pathways to appropriate treatment given the variety of side effects. Cardiometabolic effects (e.g., cardiac issues, weight gain, diabetes mellitus, hyperlipidemia); neurological effects (e.g., dizziness, seizures, sleep disturbances, movement disorders), and other side effects (e.g., sedation, behavioral activation, hyperprolactinemia, constipation)—are well-documented in adults (Abosi et al., 2018; de Filippis et al., 2023; Mazereel et al., 2020). Children may be uniquely vulnerable to pharmacological influences due to exposure at key brain and body developmental stages (Mohiuddin & Ghaziuddin, 2013) Multiple aspects of pharmacokinetics, from absorption to metabolism and excretion and withdrawal symptoms, all change during development (Lorberg et al., 2019) A 2020 systematic review of psychotropic treatment in children and adolescents (not specific to autism) found limited data about adverse events for many medications, and that long-term and rare adverse events were likely underrepresented (Solmi et al., 2020).

Non-acute mental health conditions, such as ADHD and generalized anxiety disorder, may be responsive to empirically-based psychosocial services alone or in conjunction with pharmacological treatment (Janiczak et al., 2020). We did not examine use of psychosocial services in this study, but it is advised as part of a holistic behavioral or psychosocial treatment plan (AACAP, 2015). Research on the interplay between the frequent use of psychotropic medications and behavioral supports (among other services) is urgently needed to generate and sustain a robust evidence base across intervention modalities (medications and behavioral supports) to inform families, clinicians, and policy. This study also did not examine the presence of an intellectual disability (ID) diagnosis in psychotropic prescriptions. However, prescription of psychotropics, monitoring of effectiveness and side effects, and concurrent use of psychosocial services is complicated in people with ID due at least partly to lack of provider knowledge on treating such patients (Ramerman et al., 2018). Psychosocial treatments are often developed without the inclusion of people with ID in validations studies, and they are often excluded from such treatments (Brown et al., 2011). Future work should focus on examining the impact of intellectual disability on the prescription of psychotropics and the use of psychosocial services.

This study had strengths and challenges typical of claims data analyses. National Medicaid data allow for the utilization of different categorizations of medication use, and our study years allowed for a longer period of observation than previous studies. These results do not generalize to privately insured populations. Children enrolled in Medicaid may have differences in clinical profile and complexity, or other factors such as socioeconomic status, to meet enrollment criteria. However, Medicaid coverage is common in children with autism, and half of all autistic youth may be enrolled in Medicaid (Rast et al., 2020). This study did not identify co-occurring psychiatric conditions with high specificity (e.g., types of bipolar or depression), which may limit the interpretability and translation of findings. Prescription information comes from dispensing and does not confirm medication use or compliance. Finally, while this study explored many patterns of medication use, there are others that are important to practice, including examination by race and ethnicity and other social determinants of health.

### Conclusions

Research that identifies disparities in the use of psychotropic medications across race and ethnicity, clinical characteristics, and social determinants of health points toward leveraging policy differences, such as variation in state Medicaid formularies, for understanding and addressing access barriers. This study found about half of children with autism enrolled in Medicaid had at least one psychotropic prescription, a percentage that increased with age and with the presence of co-occurring psychiatric conditions. Concurrent prescription of more than one psychotropic medication was also common, where 20% of children with autism took two or more medications for at least six months of the year. As psychotropic prescription is common in this group, understanding use, risks, and efficacy can guide research to inform safer and more effective care.

## Supplementary Information

Below is the link to the electronic supplementary material.

Supplementary file1 (DOCX 33 KB)
