# Supplementary material for: Psychotropic Medication Use in Children and Youth with Autism Enrolled in Medicaid
Source: J Autism Dev Disord. 2023 Dec 18;55(1):258–66. doi: 10.1007/s10803-023-06182-5 (PMC11228548; doi:10.1007/s10803-023-06182-5)
Supplement: Supplementary file 1 — Supplementary file1 (DOCX 33 KB) [file 10803_2023_6182_MOESM1_ESM.docx]

**Supplemental tables**

**eTable 1: ICD Codes for conditions**

| Condition | ICD-9 | ICD-10 |
| --- | --- | --- |
| Anxiety disorders | 293.84, 300.00, 300.01, 300.02, 300.09, 300.10, 300.20, 300.21, 300.22, 300.23, 300.29, 300.3, 300.5, 300.89, 300.9, 308.0, 308.1, 308.2, 308.3, 308.4, 308.9, 309.81, 313.0, 313.1, 313.21, 313.22, 313.3, 313.82, 313.83 | F06.4, F40.00, F40.01, F40.02, F40.10, F40.11, F40.210, F40.218, F40.220, F40.228, F40.230, F40.231, F40.232, F40.233, F40.240, F40.241, F40.242, F40.243, F40.248, F40.290, F40.291, F40.298, F40.8, F40.9, F41.0, F41.1, F41.3, F41.8, F41.9, F42, F42.2, F42.3, F42.4, F42.8, F42.9, F43.0, F43.10, F43.11, F43.12, F44.9, F45.8, F48.8, F48.9, F93.8, F99, R45.2, R45.5, R45.6, R45.7 |
| Depressive disorders | 296.20, 296.21, 296.22, 296.23, 296.24, 296.25, 296.26, 296.30, 296.31, 296.32, 296.33, 296.34, 296.35, 296.36, 300.4, 311 | F32.0, F32.1, F32.2, F32.3, F32.4, F32.5, F32.89, F32.9, F32.A, F33.0, F33.1, F33.2, F33.3, F33.40, F33.41, F33.42, F33.8, F33.9, F34.1 |
| Bipolar | 296.00, 296.01, 296.02, 296.03, 296.04, 296.05, 296.06, 296.10, 296.11, 296.12, 296.13, 296.14, 296.15, 296.16, 296.40, 296.41, 296.42, 296.43, 296.44, 296.45, 296.46, 296.50, 296.51, 296.52, 296.53, 296.54, 296.55, 296.56, 296.60, 296.61, 296.62, 296.63, 296.64, 296.65, 296.66, 296.7, 296.80, 296.81, 296.82, 296.89, 296.90, 296.99 | F30.10, F30.11, F30.12, F30.13, F30.2, F30.3, F30.4, F30.8, F30.9, F31.0, F31.10, F31.11, F31.12, F31.13, F31.2, F31.30, F31.31, F31.32, F31.4, F31.5, F31.60, F31.61, F31.62, F31.63, F31.64, F31.70, F31.71, F31.72, F31.73, F31.74, F31.75, F31.76, F31.77, F31.78, F31.81, F31.89, F31.9, F33.8, F34.81, F34.89, F34.9, F39 |
| ADHD, conduct disorders, and hyperkinetic syndrome | 312.00, 312.01, 312.02, 312.03, 312.10, 312.11, 312.12, 312.13, 312.20, 312.21, 312.22, 312.23, 312.30, 312.31, 312.32, 312.33, 312.34, 312.35, 312.39, 312.4, 312.81, 312.82, 312.89, 312.9, 314.00, 314.01, 314.1, 314.2, 314.8, 314.9 | F63.0, F63.1, F63.2, F63.3, F63.81, F63.89, F63.9, F90.0, F90.1, F90.2, F90.8, F90.9, F91.0, F91.1, F91.2, F91.3, F91.8, F91.9 |
| Schizophrenia or other psychotic disorders | 293.81, 293.82, 295.00, 295.01, 295.02, 295.03, 295.04, 295.05, 295.10, 295.11, 295.12, 295.13, 295.14, 295.15, 295.20, 295.21, 295.22, 295.23, 295.24, 295.25, 295.30, 295.31, 295.32, 295.33, 295.34, 295.35, 295.40, 295.41, 295.42, 295.43, 295.44, 295.45, 295.50, 295.51, 295.52, 295.53, 295.54, 295.55, 295.60, 295.61, 295.62, 295.63, 295.64, 295.65, 295.70, 295.71, 295.72, 295.73, 295.74, 295.75, 295.80, 295.81, 295.82, 295.83, 295.84, 295.85, 295.90, 295.91, 295.92, 295.93, 295.94, 295.95, 297.0, 297.1, 297.2, 297.3, 297.8, 297.9, 298.0, 298.1, 298.2, 298.3, 298.4, 298.8, 298.9 | F06.0, F06.2, F20.0, F20.1, F20.2, F20.3, F20.5, F20.81, F20.89, F20.9, F21, F22, F23, F24, F25.0, F25.1, F25.8, F25.9, F28, F29, F32.3, F33.3, F44.89 |

**eTable 2. Psychotropic medication class by co-occurring condition in 2008, 2012, 2016, and combined 2008-2016**

| **2008-2016** | | | | | |
| --- | --- | --- | --- | --- | --- |
|  | **Antidepressants** | **Neuroleptics** | **Anxiolytics / Sedatives / hypnotics** | **Stimulants** | **Anticonvulsants** |
|  | N = 290,117 | N = 316,365 | N = 44,324 | N = 299,117 | N = 217,455 |
| **Anxiety disorders** | 62.81 | 56.08 | 10.21 | 47.03 | 36.4800 |
| **Depressive disorders** | 74.08 | 65.95 | 11.27 | 48.59 | 41.15 |
| **Bipolar disorder** | 69.13 | 83.86 | 12.02 | 56.70 | 50.78 |
| **ADHD, conduct disorders, and hyperkinetic syndrome** | 44.75 | 51.10 | 6.68 | 58.09 | 29.12 |
| **Schizophrenia or other psychotic disorders** | 71.47 | 88.37 | 13.77 | 46.42 | 55.73 |
| **None of these** | 9.57 | 11.05 | 1.63 | 5.54 | 14.10 |
| **2008** | | | | | |
|  | **Antidepressants** | **Neuroleptics** | **Anxiolytics / Sedatives / hypnotics** | **Stimulants** | **Anticonvulsants** |
|  | N = 27,435 | N = 48,129 | N = 3,921 | N = 25,384 | N = 22,832 |
| **Anxiety disorders** | 53.00 | 62.02 | 6.89 | 28.56 | 28.55 |
| **Depressive disorders** | 61.18 | 67.04 | 6.51 | 28.69 | 30.32 |
| **Bipolar disorder** | 48.58 | 85.88 | 6.71 | 35.45 | 40.06 |
| **ADHD, conduct disorders, and hyperkinetic syndrome** | 32.18 | 58.38 | 4.06 | 45.31 | 21.69 |
| **Schizophrenia or other psychotic disorders** | 48.16 | 88.03 | 8.23 | 22.29 | 42.89 |
| **None of these** | 12.71 | 23.67 | 2.08 | 8.70 | 13.73 |
| **2012** | | | | | |
|  | **Antidepressants** | **Neuroleptics** | **Anxiolytics / Sedatives / hypnotics** | **Stimulants** | **Anticonvulsants** |
|  | N = 50,658 | N = 75,549 | N = 5,082 | N = 52,834 | N = 38,329 |
| **Anxiety disorders** | 51.21 | 50.69 | 5.13 | 30.66 | 24.15 |
| **Depressive disorders** | 62.11 | 58.49 | 5.35 | 29.63 | 27.30 |
| **Bipolar disorder** | 50.03 | 79.43 | 5.33 | 36.85 | 36.04 |
| **ADHD, conduct disorders, and hyperkinetic syndrome** | 31.10 | 48.42 | 2.73 | 48.31 | 18.30 |
| **Schizophrenia or other psychotic disorders** | 51.13 | 83.77 | 6.49 | 26.17 | 39.82 |
| **None of these** | 10.20 | 16.51 | 1.17 | 7.56 | 11.38 |
| **2016** | | | | | |
|  | **Antidepressants** | **Neuroleptics** | **Anxiolytics / Sedatives / hypnotics** | **Stimulants** | **Anticonvulsants** |
|  | N = 85,243 | N = 97,191 | N = 8,104 | N = 86,062 | N = 58,328 |
| **Anxiety disorders** | 53.59 | 41.87 | 5.76 | 30.06 | 21.91 |
| **Depressive disorders** | 65.66 | 51.32 | 6.04 | 29.45 | 24.89 |
| **Bipolar disorder** | 52.66 | 75.82 | 6.22 | 35.40 | 36.62 |
| **ADHD, conduct disorders, and hyperkinetic syndrome** | 32.44 | 39.22 | 2.96 | 47.85 | 17.10 |
| **Schizophrenia or other psychotic disorders** | 58.34 | 81.94 | 6.83 | 23.31 | 37.25 |
| **None of these** | 8.55 | 10.63 | 0.80 | 5.82 | 9.95 |

**eTable 3. Top 10 psychotropic medications prescribed to children with autism ages 0-2 years and percentage with epilepsy diagnosis, 2016**

| Age years | Meds | N of Children | Co-occurring epilepsy | |
| --- | --- | --- | --- | --- |
|  |  |  | N | % |
| 0 | * | * | * | * |
| 1 | Levetiracetam | 27 | 25 | 92.59 |
| 1 | Diazepam | 19 | 14 | 73.68 |
| * | * | * | * | * |
| 2 | Levetiracetam | 146 | 131 | 89.73 |
| 2 | Diazepam | 118 | 91 | 77.12 |
| 2 | Oxcarbazepine | 47 | 41 | 87.23 |
| 2 | Clonazepam | 30 | 22 | 73.33 |
| 2 | Phenobarbital | 28 | 24 | 85.71 |
| 2 | Clobazam | 28 | 27 | 96.43 |
| 2 | Topiramate | 26 | 24 | 92.31 |
| 2 | Risperidone | 22 | . | . |
| 2 | Lamotrigine | 14 | 13 | 92.86 |
| 2 | Zonisamide | 11 | 11 | 100.00 |

*Censored due to small cell size

**eTable 4. Factors associated with psychotropic medication use in 2008, 2012 and 2016**

|  | **Any psychotropic medication** | | | | | |
| --- | --- | --- | --- | --- | --- | --- |
|  | **2008** | | **2012** | | **2016** | |
|  | **RR** | **95% CI** | **RR** | **95% CI** | **RR** | **95% CI** |
| **Age** |  |  |  |  |  |  |
| 0-5 | 0.42 | (0.41,0.43) | 0.33 | (0.33,0.34) | 0.25 | (0.24,0.25) |
| 6-11 | 0.83 | (0.82,0.83) | 0.79 | (0.79,0.80) | 0.75 | (0.74,0.75) |
| 12-17 (ref) |  | |  | |  | |
| 18-21 | 1.09 | (1.08,1.10) | 1.11 | (1.10,1.12) | 1.15 | (1.14,1.16) |
| **Sex** |  |  |  |  |  |  |
| Female | 0.98 | (0.97,0.99) | 1.01 | (1.00,1.02) | 1.03 | (1.02,1.03) |
| Male |  | |  | |  | |
| **Race** |  |  |  |  |  |  |
| White (ref) |  | |  | |  | |
| Black | 0.85 | (0.84,0.86) | 0.87 | (0.86,0.88) | 0.88 | (0.87,0.89) |
| Asian/Hawaiian/Pacific Islander | 0.73 | (0.70,0.77) | 0.68 | (0.66,0.70) | 0.68 | (0.66,0.69) |
| Hispanic/Latino | 0.85 | (0.84,0.87) | 0.81 | (0.81,0.82) | 0.79 | (0.79,0.8) |
| Multiracial | 1.03 | (0.99,1.07) | 1.02 | (1.00,1.05) | 1.04 | (1.01,1.06) |
| American Indian and Alaska Native, non-Hispanic | 0.88 | (0.84,0.92) | 0.88 | (0.84,0.91) | 0.84 | (0.81,0.87) |
| Missing | 0.94 | (0.92,0.95) | 0.96 | (0.95,0.97) | 0.91 | (0.9,0.92) |
| **Eligibility** |  |  |  |  |  |  |
| Poverty | 0.81 | (0.80,0.82) | 0.89 | (0.88,0.89) | 0.92 | (0.91,0.93) |
| Disability (ref) |  |  |  |  |  |  |
| Other | 0.95 | (0.94,0.97) | 1.02 | (1.01,1.03) | 0.95 | (0.94,0.95) |
| Missing | - | - | - | - | 1.01 | (0.97,1.05) |
| **Urbanicity** |  |  |  |  |  |  |
| Urban (ref) |  | |  | |  | |
| Suburban | 1.07 | (1.05,1.08) | 1.11 | (1.10,1.12) | 1.09 | (1.09,1.1) |
| Rural | 1.05 | (1.02,1.07) | 1.09 | (1.08,1.11) | 1.07 | (1.06,1.09) |
| Missing | 0.91 | (0.86,0.96) | 1.01 | (0.95,1.07) | 0.99 | (0.96,1.01) |
| **Co-occurring diagnoses** |  |  |  |  |  |  |
| Anxiety disorders | 1.17 | (1.16,1.19) | 1.19 | (1.18,1.20) | 1.27 | (1.26,1.27) |
| Depressive disorders | 1.08 | (1.06,1.09) | 1.07 | (1.06,1.09) | 1.08 | (1.07,1.09) |
| Bipolar disorder | 1.27 | (1.26,1.28) | 1.29 | (1.29,1.30) | 1.24 | (1.23,1.24) |
| ADHD, conduct disorders, and hyperkinetic syndrome | 1.68 | (1.67,1.70) | 1.88 | (1.87,1.89) | 2.09 | (2.08,2.1) |
| Schizophrenia or other psychotic disorders | 1.13 | (1.11,1.14) | 1.11 | (1.09,1.12) | 1.12 | (1.11,1.13) |

**eTable 5. Factors associated with polypharmacy use in 2008, 2012 and 2016**

|  | **Polypharmacy** | | | | | |
| --- | --- | --- | --- | --- | --- | --- |
|  | **2008** | | **2012** | | **2016** | |
|  | **RR** | **95% CI** | **RR** | **95% CI** | **RR** | **95% CI** |
| **Age** |  |  |  |  |  |  |
| 0-5 | 0.07 | (0.06,0.08) | 0.06 | (0.05,0.07) | 0.05 | (0.04,0.05) |
| 6-11 | 0.47 | (0.45,0.49) | 0.43 | (0.42,0.45) | 0.41 | (0.4,0.42) |
| 12-17 |  | |  | |  | |
| 18-21 | 1.33 | (1.27,1.4) | 1.40 | (1.35,1.44) | 1.52 | (1.48,1.56) |
| **Sex** |  |  |  |  |  |  |
| Female | 1.06 | (1.02,1.11) | 1.12 | (1.08,1.16) | 1.16 | (1.12,1.19) |
| Male |  | |  | |  | |
| **Race** |  |  |  |  |  |  |
| White |  | |  | |  | |
| Black | 0.59 | (0.56,0.63) | 0.67 | (0.64,0.7) | 0.64 | (0.62,0.67) |
| Asian/Hawaiian/Pacific Islander | 0.55 | (0.45,0.66) | 0.47 | (0.41,0.54) | 0.42 | (0.38,0.47) |
| Hispanic/Latino | 0.64 | (0.59,0.69) | 0.59 | (0.56,0.62) | 0.55 | (0.53,0.58) |
| Multiracial | 1.00 | (0.84,1.18) | 1.06 | (0.96,1.17) | 1.01 | (0.93,1.1) |
| American Indian and Alaska Native, non-Hispanic | 0.86 | (0.71,1.03) | 0.84 | (0.73,0.97) | 0.76 | (0.67,0.87) |
| Missing | 0.94 | (0.88,1) | 0.91 | (0.87,0.96) | 0.80 | (0.77,0.83) |
| **Eligibility** |  |  |  |  |  |  |
| Poverty | 0.57 | (0.53,0.61) | 0.65 | (0.62,0.67) | 0.74 | (0.72,0.76) |
| Disability |  |  |  |  |  |  |
| Other | 0.95 | (0.89,1) | 1.09 | (1.04,1.13) | 0.90 | (0.87,0.93) |
| Missing | - | - | - | - | 1.30 | (1.13,1.5) |
| **Urbanicity** |  |  |  |  |  |  |
| Urban |  | |  | |  | |
| Suburban | 1.14 | (1.09,1.19) | 1.21 | (1.17,1.24) | 1.17 | (1.14,1.21) |
| Rural | 1.14 | (1.05,1.23) | 1.14 | (1.07,1.21) | 1.23 | (1.17,1.3) |
| Missing | 0.83 | (0.67,1.04) | 0.99 | (0.78,1.24) | 0.82 | (0.74,0.91) |
| **Co-occurring diagnoses** |  |  |  |  |  |  |
| Anxiety disorders | 1.30 | (1.23,1.37) | 1.34 | (1.29,1.39) | 1.41 | (1.37,1.45) |
| Depressive disorders | 1.20 | (1.13,1.28) | 1.02 | (0.98,1.07) | 1.06 | (1.02,1.1) |
| Bipolar disorder | 2.03 | (1.94,2.13) | 2.14 | (2.07,2.21) | 2.04 | (1.98,2.1) |
| ADHD, conduct disorders, and hyperkinetic syndrome | 1.83 | (1.76,1.91) | 1.96 | (1.9,2.02) | 2.19 | (2.13,2.24) |
| Schizophrenia or other psychotic disorders | 1.21 | (1.13,1.3) | 1.19 | (1.13,1.25) | 1.28 | (1.23,1.34) |
